# Assessing the age- and gender-dependence of the severity and case fatality rates of COVID-19 disease in Spain

**DOI:** 10.12688/wellcomeopenres.15996.1

**Published:** 2020-06-02

**Authors:** Paula Moraga, David I. Ketcheson, Hernando C. Ombao, Carlos M. Duarte

**Affiliations:** 1 Department of Mathematical Sciences, University of Bath, Bath, Somerset, BA2 7AY, UK; 2 Computer, Electrical, and Mathematical Sciences & Engineering Division, King Abdullah University of Science and Technology (KAUST), Thuwal, Saudi Arabia; 3 Biological and Environmental Science and Engineering Division, King Abdullah University of Science and Technology (KAUST), Thuwal, Saudi Arabia

**Keywords:** COVID-19, epidemics, mortality, case fatality rate, age, gender

## Abstract

**Background:** The assessment of the severity and case fatality rates of coronavirus disease 2019 (COVID-19) and the determinants of its variation is essential for planning health resources and responding to the pandemic. The interpretation of case fatality rates (CFRs) remains a challenge due to different biases associated with surveillance and reporting. For example, rates may be affected by preferential ascertainment of severe cases and time delay from disease onset to death. Using data from Spain, we demonstrate how some of these biases may be corrected when estimating severity and case fatality rates by age group and gender, and identify issues that may affect the correct interpretation of the results.

**Methods:** Crude CFRs are estimated by dividing the total number of deaths by the total number of confirmed cases. CFRs adjusted for preferential ascertainment of severe cases are obtained by assuming a uniform attack rate in all population groups, and using demography-adjusted under-ascertainment rates. CFRs adjusted for the delay between disease onset and death are estimated by using as denominator the number of cases that could have a clinical outcome by the time rates are calculated. A sensitivity analysis is carried out to compare CFRs obtained using different levels of ascertainment and different distributions for the time from disease onset to death.

**Results:** COVID-19 outcomes are highly influenced by age and gender. Different assumptions yield different CFR values but in all scenarios CFRs are higher in old ages and males.

**Conclusions:** The procedures used to obtain the CFR estimates require strong assumptions and although the interpretation of their magnitude should be treated with caution, the differences observed by age and gender are fundamental underpinnings to inform decision-making.

## Introduction

The coronavirus disease 2019 (COVID-19) has spread to nearly every country in the world since it first emerged in the Hubei province of China in 2019. As of 14 May 2020, more than 4.22 million cases and more than 290,000 deaths have been reported worldwide ^1^. While people of any age may get infected, COVID-19 symptoms are particularly severe for the elderly and those with underlying health conditions, which creates a disproportionate risk and need for intensive care in these groups. Understanding the severity of the disease in the different population groups is essential to help predict the demand of healthcare resources and to design effective mitigation policies.

Case fatality rates (CFR) are often used to characterize the severity of the disease. The crude CFR is obtained by dividing the cumulative number of deaths by the cumulative number of reported cases. This indicator is simple to calculate but is difficult to interpret due to different biases ^2^. First, the clinical outcome (recovery or death) of the most recent cases may be unknown due to the delay between disease onset and death which may underestimate the true CFR. Moreover, limited capabilities in testing result in most of people tested being only those with the most severe symptoms and most likely to experience fatal outcomes. As a result, crude CFRs may overestimate rates that are defined based on the actual number of infected people (including those with weak or no symptoms).

Crude fatality rates can be adjusted in a number of ways to obtain estimates that more accurately represent the severity of the disease in each of the population groups. For example, censoring can be taken into account by using the distribution of the time between disease onset and death to determine the number of cases that could experience an outcome by the point in time when the rates are calculated. The under-ascertainment of COVID-19 in different groups can also be corrected by using the population demographics.

Here, we calculate crude and adjusted fatality rates by age group and gender in Spain. Spain is one of the hardest-hit countries in the pandemic with 272646 cases and 27321 deaths as of 14 May 2020. The country is characterized by one of the longest life expectancies and lowest birth rates in the world ^3^ and, thus, has a large percentage of older adults. Moreover, it is characterized by a sociable lifestyle and extensive inter-generational interactions which may accelerate the spread of the virus. Accurate assessment of CFRs by age group and gender is essential to help planning responses that help save lives.

First, we present the data on population, confirmed cases and deaths of Spain. We then demonstrate how to calculate crude and adjusted CFRs by population group and present the estimates for Spain. We discuss the limitations of the methods and conduct a sensitivity analysis where we compare CFRs adjusted under different assumptions.

## Methods

### Data

**Figure 1.**
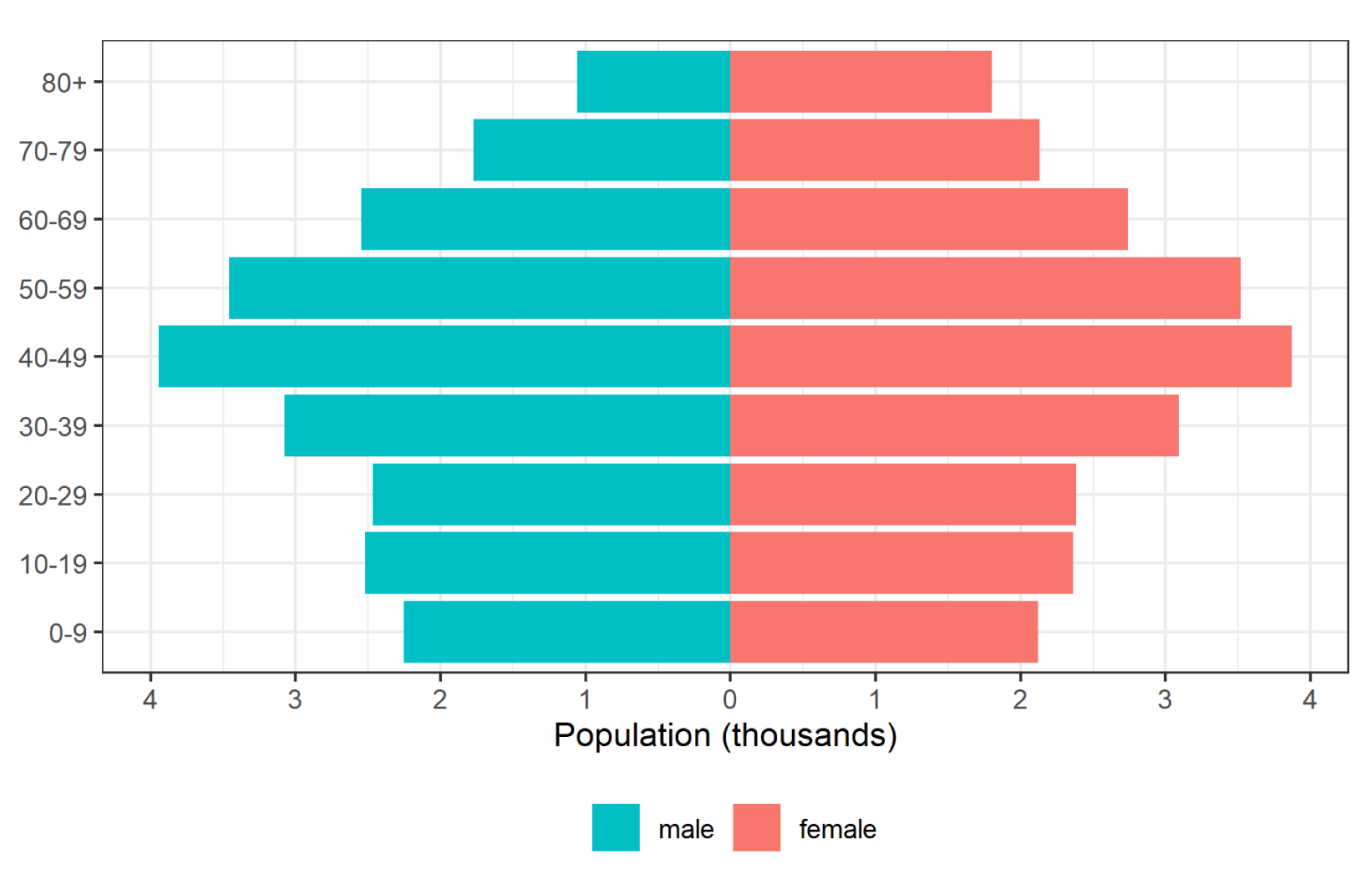
Population by age group and gender in Spain, 2019.

Population data for Spain stratified by age group and gender for 2019 are obtained from the National Institute of Statistics of Spain ^4^ ( Figure 1). We note the large percentage of older adults with over-60 males and females representing 11.41% and 14.16% of the whole population, respectively.

**Figure 2.**
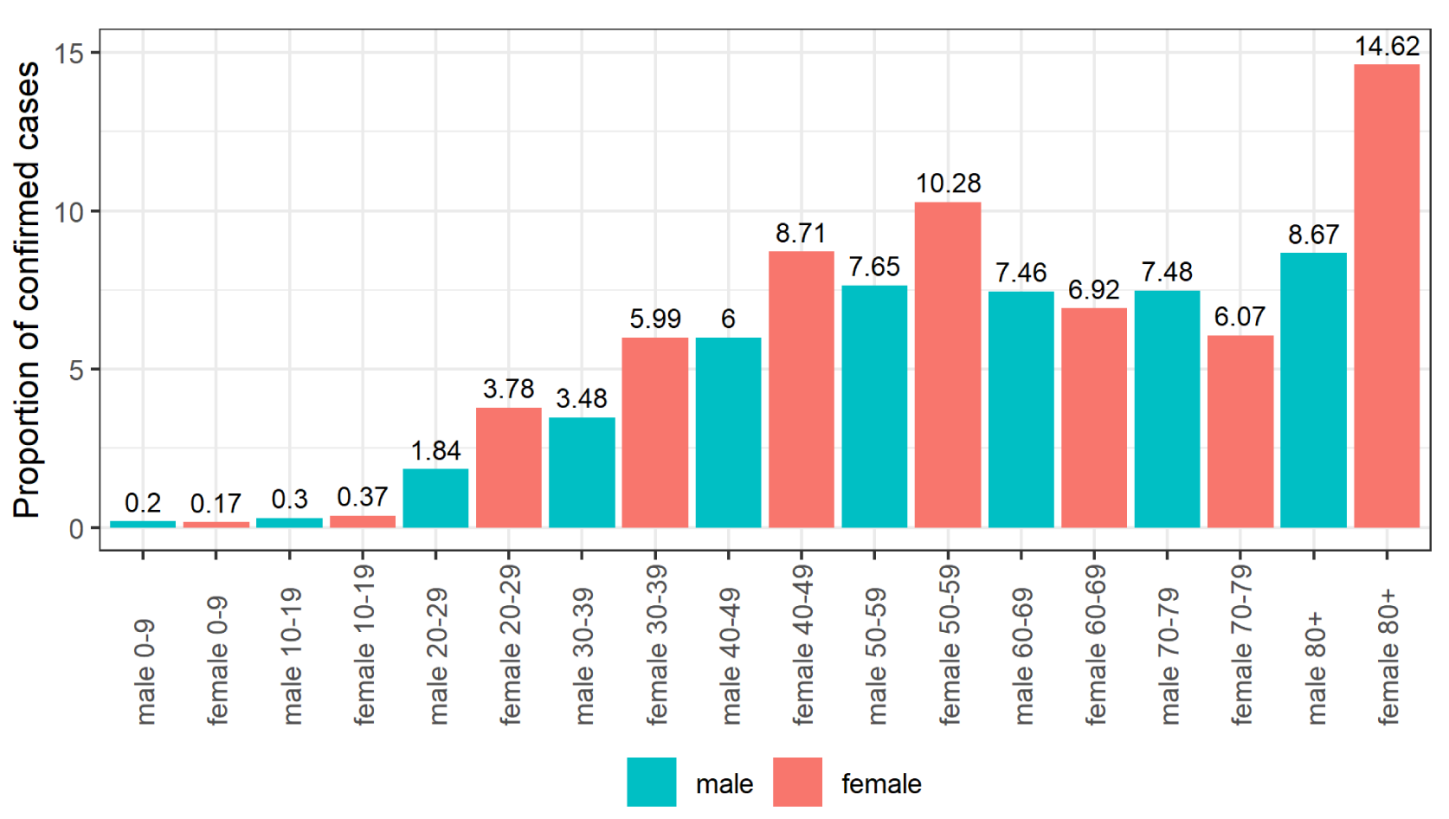
Proportion of confirmed cases by age group and gender.

**Figure 3.**
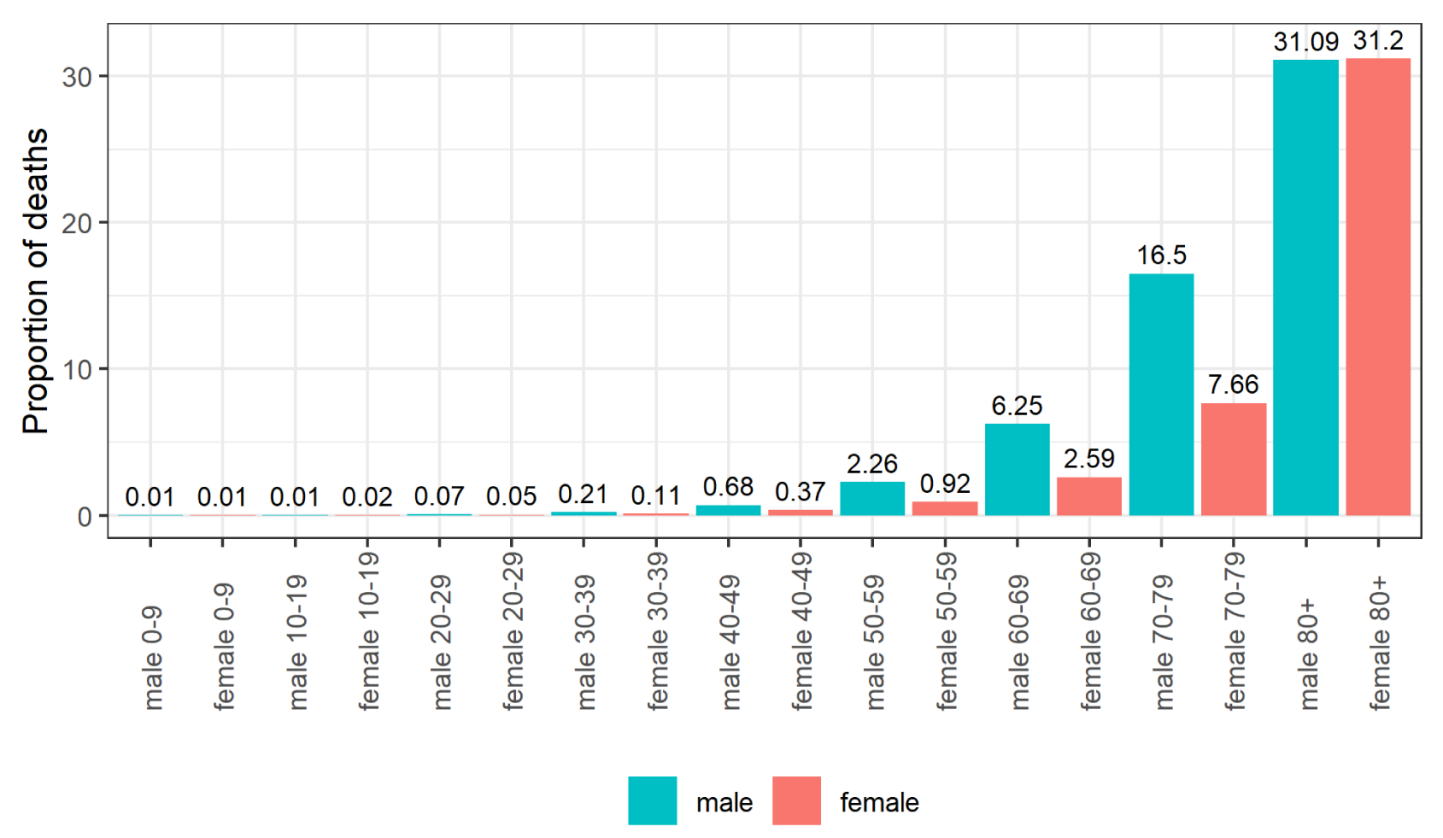
Proportion of deaths by age group and gender.

**Figure 4.**
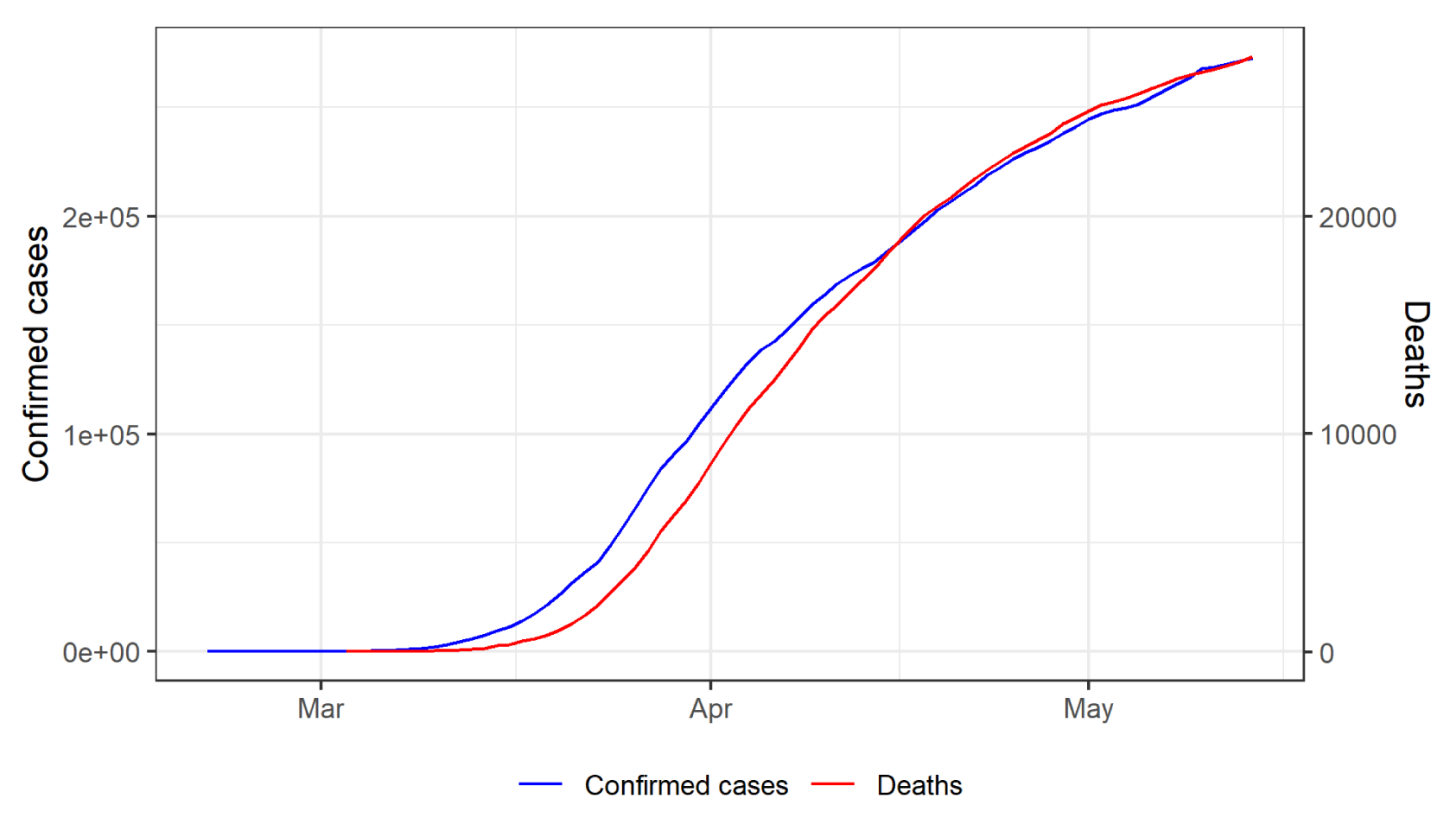
Cumulative total number of confirmed cases and deaths over time.

Data on the daily total confirmed cases and deaths, as well as daily confirmed cases and deaths by age group and gender from a subset of the population are reported by the Spanish Ministry of Health and provided by 5. Assuming this subset is representative of all cases, in terms of the relative distribution among age group and gender, we can estimate the daily number of confirmed cases in each group by multiplying the total number of cases by the proportion of cases in each group. Daily number of deaths in each age group and gender are calculated following a similar procedure. Figure 2 and Figure 3 show the proportion of cases and deaths, respectively, in each age group and gender. We observe a low proportion of confirmed cases in young people (under 20 years old) and a high proportion of deaths in older age groups and males. Figure 4 shows the total number of confirmed cases and deaths over time.

### Relative risk

We can examine the relative risks in each age group and gender to compare the severity of the disease between population groups. The relative risk in each population group is obtained by dividing the number of deaths in a group by the total population in that group, and normalizing the values so the risk of males older than 80 is equal to 1. We observe a roughly tenfold increase in risk for every 20 year increase in age, consistent with an earlier smaller study of cases in China ^6^.

### Case fatality rate

At any point in time, the crude CFR is calculated by dividing the cumulative number of deaths by the cumulative number of reported cases. As noted, CFRs may be affected by preferential ascertainment of severe cases. This is likely to occur in COVID-19 where cases asymptomatic or with mild symptoms are less likely to seek medical care or be included in the surveillance data. This could result in an upward bias (or overestimate) of the crude CFRs by under-reporting of cases. We can partially correct this bias by calculating the adjusted daily number of confirmed cases following the procedure detailed in 6. Specifically, we calculate NC _*a*_ = pop _*a*_/cases _*a*_ where pop _*a*_ and cases _*a*_ are the population and the number of cases, respectively, in group *a*, *a* ∈ { males 0–9, males 10–19, males 20–29, males 30–39, males 40–49, males 50–59, males 60–69, males 70–79, males 80+, females 0–9, females 10–19, females 20–29, females 30–39, females 40–49, females 50–59, females 60–69, females 70–79, females 80+ }. We assume perfect ascertainment in the group with maximum 1/NC _*a*_ value which is the group of males older than 80. Then, we assume the attack rate is the same in all groups and estimate the adjusted number of cases in each population group by multiplying the confirmed cases by NC _*a*_/NC _males 80+_. Figure 6 and Figure 7 show the cumulative confirmed cases and the cases adjusted for preferential ascertainment over time for each age group and gender. Finally, we calculate the CFRs adjusted for preferential ascertainment by dividing the cumulative number of deaths by the cumulative number of adjusted cases in each population group. We also calculate 95% confidence intervals using exact binomial tests ^7^.

**Figure 5.**
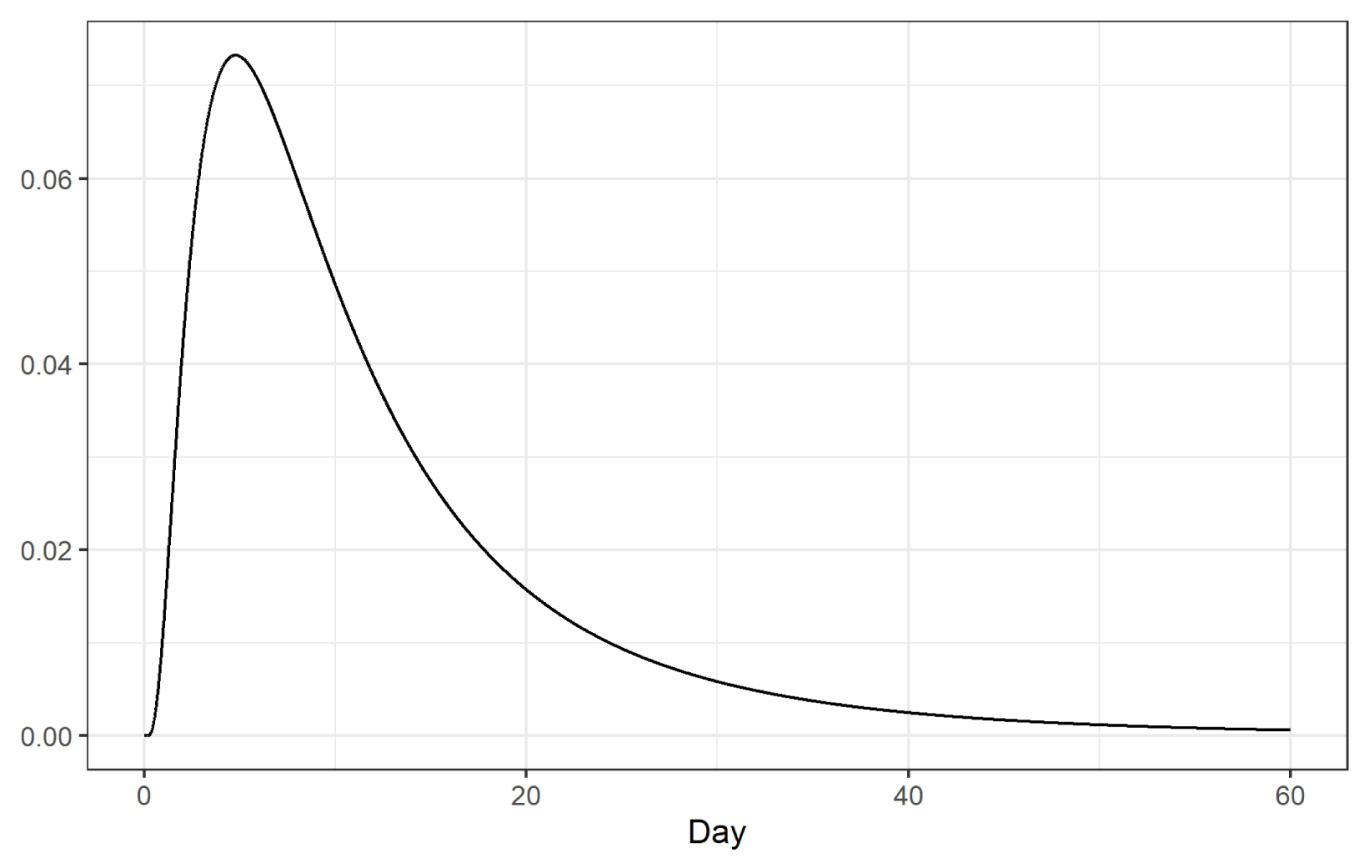
Log-normal distribution of the time from disease onset to death with mean 13 days and standard deviation 12.7 days.

**Figure 6.**
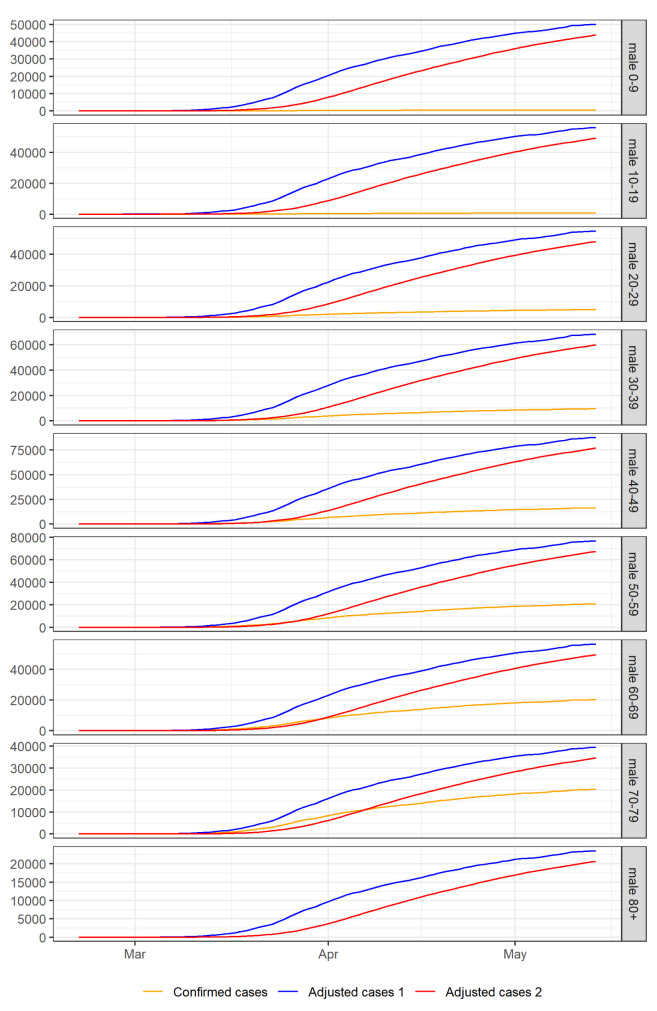
Cumulative confirmed and adjusted cases over time for each age group in males. Adjusted cases 1 are cases adjusted for preferential ascertainment of severe cases. Adjusted cases 2 are cases adjusted for preferential ascertainment of severe cases and time delay between confirmation and death.

**Figure 7.**
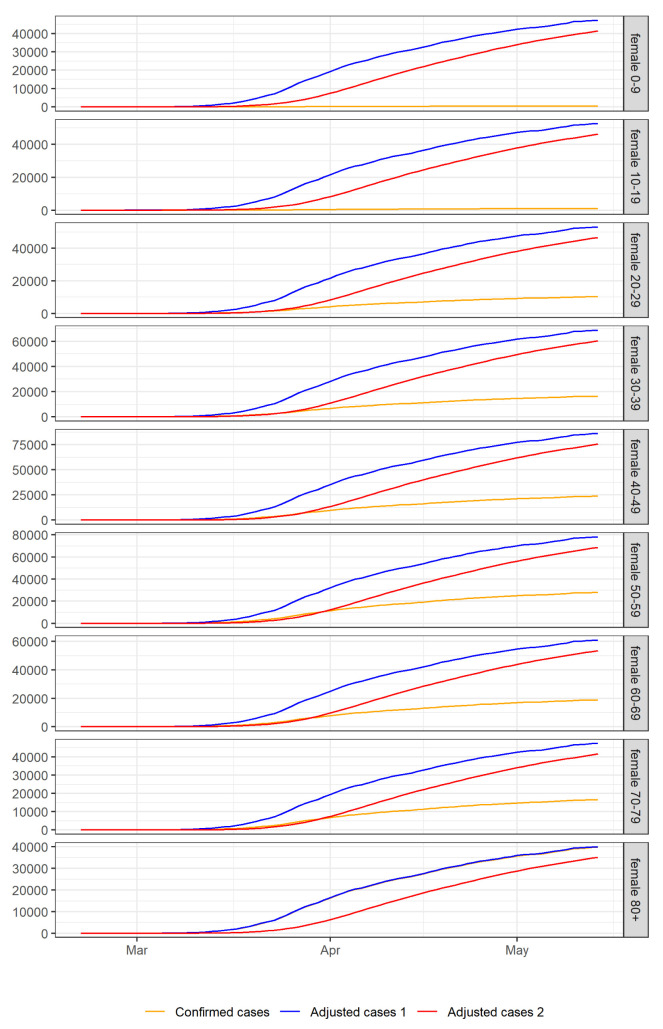
Cumulative confirmed and adjusted cases over time for each age group in females. Adjusted cases 1 are cases adjusted for preferential ascertainment of severe cases. Adjusted cases 2 are cases adjusted for preferential ascertainment of severe cases and time delay between confirmation and death.

CFRs can also be biased due to the delay between disease onset and death. At any moment in time, the cumulative number of confirmed cases includes people who have not yet died but may do so in the future. Therefore, crude fatality rates may underestimate the true severity of the disease. We can correct this bias by replacing the denominator with an estimate of the cumulative number of cases with known outcomes by the time rates are calculated. Specifically, we adjust for this bias as follows. Let *T* be the point in time when the CFRs are calculated. The probability that a case confirmed at time *t*, *t* = 1, . . . , *T*, has a known outcome by time *T* is expressed as $p_{t}=\int_{m=0}^{T-t}g(m)dm,$ where *g*( *m*) denotes the probability density that a case has a known outcome *m* days after the disease onset. The cumulative number of cases in group *a* with known outcomes by time *T* can be calculated as

$$\text{case}{\text{s}}_{a}=\sum_{t=1}^{T}\text{ncase}{\text{s}}_{at}\times p_{t,}$$

where ncases _*at*_ denotes the new number of cases in population group *a* and date *t*. Here we calculate the number of adjusted cases assuming a log-normal distribution of the time from disease onset to death with mean equal to 13 days and a standard deviation equal to 12.7 ^8^ ( Figure 5). Figure 6 and Figure 7 show cumulative cases adjusted for preferential ascertainment of severe cases and time delay between confirmation and death for each population group. Then we calculate corrected CFRs using the adjusted cases as denominator and 95% confidence intervals using an exact binomial test.

### Sensitivity analysis

The procedure we used to obtain adjusted CFRs requires strong assumptions that could greatly affect the results. First, we have adjusted crude CFRs by preferential ascertainment of severe cases by assuming complete ascertainment in the group with the highest attack rates (males older than 80). We then have assumed a uniform attack rate in all population groups, and used demography-adjusted under-ascertainment rates to obtain estimates of the number of infected individuals in each population group. However, there could also be under-ascertainment in the males older than 80 group due to extensive strain on the health system, and this fact could mean the CFR estimates are only an upper bound on the real values. We could correct this bias by further scaling the number of cases after the initial demographic adjustment. For example, we could multiply the adjusted cases by a value *α* > 1 to obtain a higher number of infected cases and lower CFRs. Moreover, the uniform attack rate assumption could be incorrect if certain population groups have more interactions with other people and are more exposed to the disease.

CFRs may also be biased due to the delay between disease onset and death. To correct this bias, we have considered a log-normal distribution with mean 13 days and standard deviation 12.7 days for the time from disease onset to death ^8^, and estimated the CFRs using as denominator the cumulative number of cases that could have a clinical outcome by the time rates are calculated. However, other distributions may be considered that could change the results.

To illustrate these limitations, we conduct a sensitivity analysis where we calculate the CFRs using different levels of ascertainment and different distributions for the time from disease onset to death. Specifically, we estimate the adjusted number of cases in each population group by multiplying the confirmed cases by NC _*a*_/NC _males 80+_× *α* using *α* values equal to 1, 1.5 and 2. We also use delay distributions equal to a log-normal distribution with mean 13 days and standard deviation 12.7 days ^8^ ( Figure 5), and a Gamma with mean 18.8 days and coefficient of variation 0.45 days ^9^ ( Figure 9).

**Figure 8.**
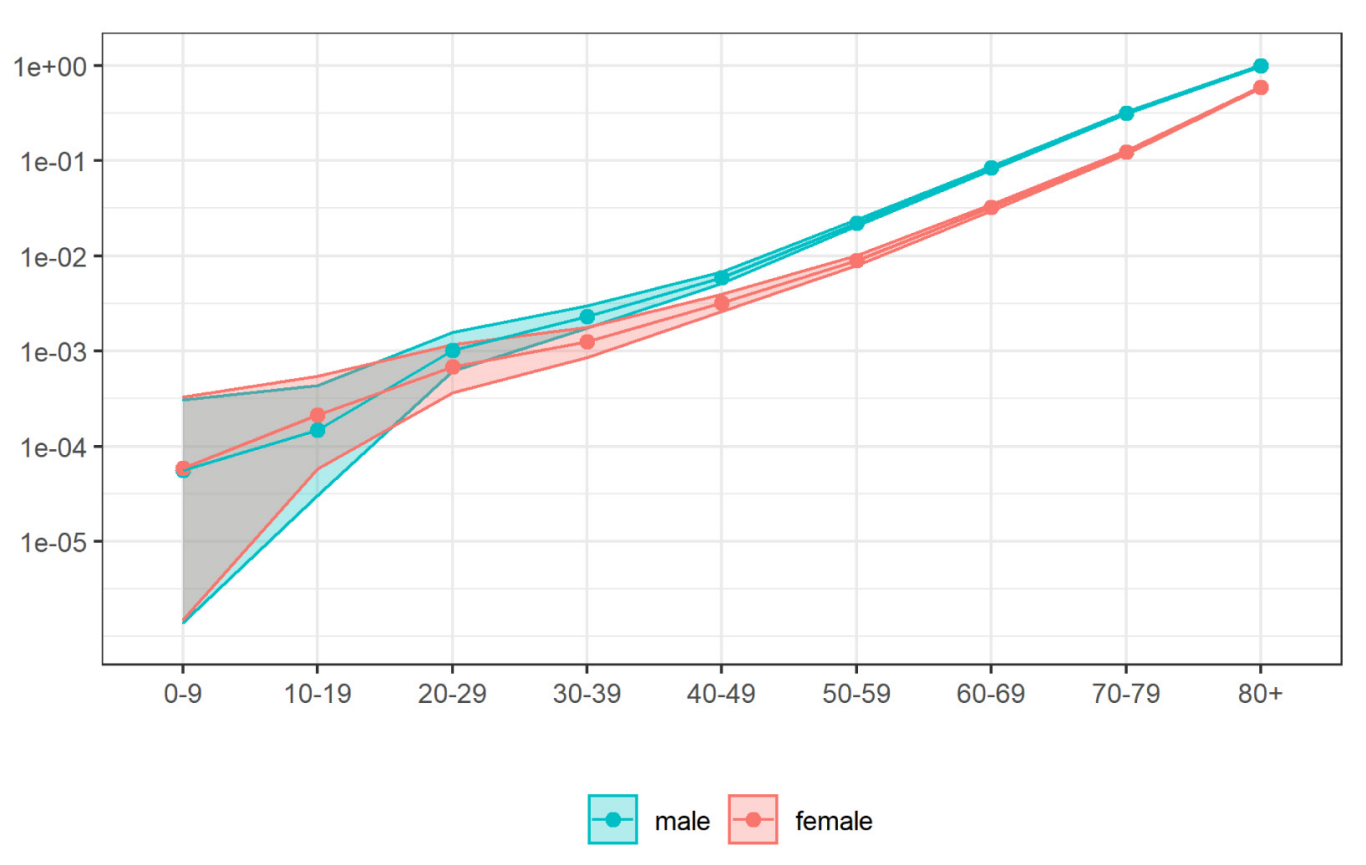
Relative risk of each population group.

**Figure 9.**
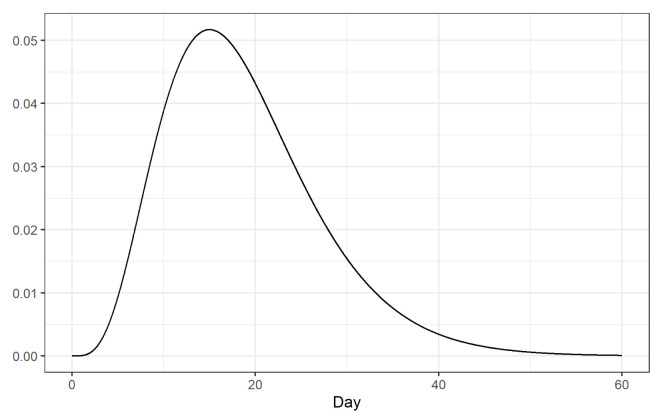
Gamma distribution of the time from disease onset to death with mean 18.8 days and coefficient of variation 0.45 days.

### Analysis

Analysis are performed with the statistical software R version 3.6.1 ^10^. Plots are created with the R package ggplot2 version 3.3.0 ^11^.

## Results

Figure 8 shows the relative risks in each age group and gender. We note the risk of COVID-19 increases with age and is higher for males than for females for all age groups except 0–9 and 10–19. Table 1 shows the crude and adjusted CFRs by age group and gender calculated on 14 May 2020. This table also shows the CFRs by age group obtained from aggregated time series of cases in mainland China by Verity *et al.* ^6^. We observe CFRs are much higher in age groups older than 60 and, for most age groups, in males. We observe the adjusted CFRs obtained with the data from Spain are smaller than the CFRs obtained by Verity *et al.* ^6^ for all except the oldest two groups, and the confidence intervals for the CFRs of Spain are much smaller due to the use of a larger dataset. Table 2 shows the CFRs estimated under different scenarios assuming different levels of ascertainment and distributions for the time from disease onset to death. We observe that in all scenarios CFRs are higher in older age groups and males but yield different values for the CFRs.

**Table 1.** Crude and adjusted case fatality rates for preferential ascertainment and time delay between disease onset and death. Adjusted 1 are rates adjusted for preferential ascertainment of severe cases. Adjusted 2 are rates adjusted for preferential ascertainment of severe cases and time delay between confirmation and death. Verity *et al.* estimates are rates obtained from aggregate time series of cases in mainland China ^6^.

Group	Crude	Adjusted 1	Adjusted 2	Verity *et al.*
male 0–9	0.187 (0.00474, 1.04)	0.00199 (5.04e-05, 0.0111)	0.00227 (5.75e-05, 0.0127)	0.0026 (0.000312, 0.0382)
female 0–9	0.217 (0.0055, 1.21)	0.00212 (5.36e-05, 0.0118)	0.00241 (6.11e-05, 0.0135)	
male 10–19	0.361 (0.0746, 1.05)	0.00534 (0.0011, 0.0156)	0.00609 (0.00126, 0.0178)	0.0148 (0.00288, 0.0759)
female 10–19	0.394 (0.107, 1)	0.00759 (0.00207, 0.0194)	0.00866 (0.00236, 0.0222)	
male 20–29	0.399 (0.244, 0.616)	0.0364 (0.0222, 0.0562)	0.0415 (0.0254, 0.0641)	0.06 (0.0317, 0.132)
female 20–29	0.126 (0.0671, 0.215)	0.0245 (0.013, 0.0418)	0.0279 (0.0149, 0.0477)	
male 30–39	0.601 (0.456, 0.778)	0.0831 (0.0629, 0.108)	0.0948 (0.0718, 0.123)	0.146 (0.103, 0.255)
female 30-39	0.19 (0.129, 0.269)	0.045 (0.0306, 0.0638)	0.0513 (0.0349, 0.0728)	
male 40–49	1.14 (0.986, 1.32)	0.213 (0.183, 0.245)	0.243 (0.209, 0.28)	0.295 (0.221, 0.422)
female 40-49	0.421 (0.343, 0.512)	0.116 (0.0943, 0.141)	0.132 (0.108, 0.161)	
male 50–59	2.96 (2.74, 3.2)	0.802 (0.74, 0.867)	0.915 (0.844, 0.989)	1.25 (1.03, 1.55)
female 50-59	0.899 (0.792, 1.02)	0.321 (0.283, 0.364)	0.367 (0.323, 0.415)	
male 60–69	8.39 (8.02, 8.78)	3.01 (2.87, 3.15)	3.43 (3.28, 3.6)	3.99 (3.41, 4.55)
female 60–69	3.75 (3.48, 4.03)	1.16 (1.07, 1.25)	1.32 (1.23, 1.42)	
male 70–79	22.1 (21.5, 22.7)	11.4 (11.1, 11.7)	13 (12.7, 13.4)	8.61 (7.48, 9.99)
female 70–79	12.7 (12.1, 13.2)	4.41 (4.23, 4.6)	5.03 (4.83, 5.25)	
male 80+	35.9 (35.3, 36.5)	35.9 (35.3, 36.5)	41 (40.3, 41.7)	13.4 (11.2, 15.9)
female 80+	21.4 (21, 21.8)	21.2 (20.8, 21.6)	24.2 (23.8, 24.7)	

**Table 2.** Case fatality rates adjusted for preferential ascertainment and time delay between disease onset and death under different scenarios. Scaling values *α* are set to 1, 1.5 and 2, and delay distributions are equal to a log-normal distribution with mean 13 days and standard deviation 12.7 days or a Gamma with mean 18.8 days and coefficient of variation 0.45 days.

Distribution *α*	Log-normal 1	Log-normal 1.5	Log-normal 2	Gamma 1	Gamma 1.5	Gamma 2
male 0–9	0.00227 (5.75e- 05, 0.0127)	0.00151 (3.84e-05, 0.00844)	0.00114 (2.88e-05, 0.00633)	0.00245 (6.19e- 05, 0.0136)	0.00163 (4.13e-05, 0.00909)	0.00122 (3.1e- 05, 0.00682)
female 0–9	0.00241 (6.11e- 05, 0.0135)	0.00161 (4.07e-05, 0.00897)	0.00121 (3.06e-05, 0.00673)	0.0026 (6.58e- 05, 0.0145)	0.00173 (4.39e-05, 0.00965)	0.0013 (3.29e- 05, 0.00724)
male 10–19	0.00609 (0.00126, 0.0178)	0.00406 (0.000837, 0.0119)	0.00304 (0.000628, 0.0089)	0.00656 (0.00135, 0.0192)	0.00437 (0.000901, 0.0128)	0.00328 (0.000676, 0.00958)
female 10–19	0.00866 (0.00236, 0.0222)	0.00577 (0.00157, 0.0148)	0.00433 (0.00118, 0.0111)	0.00933 (0.00254, 0.0239)	0.00622 (0.00169, 0.0159)	0.00466 (0.00127, 0.0119)
male 20–29	0.0415 (0.0254, 0.0641)	0.0277 (0.0169, 0.0427)	0.0208 (0.0127, 0.0321)	0.0447 (0.0273, 0.069)	0.0298 (0.0182, 0.046)	0.0224 (0.0137, 0.0345)
female 20–29	0.0279 (0.0149, 0.0477)	0.0186 (0.00991, 0.0318)	0.014 (0.00743, 0.0239)	0.03 (0.016, 0.0514)	0.02 (0.0107, 0.0343)	0.015 (0.008, 0.0257)
male 30–39	0.0948 (0.0718, 0.123)	0.0632 (0.0479, 0.0819)	0.0474 (0.0359, 0.0614)	0.102 (0.0773, 0.132)	0.0681 (0.0515, 0.0882)	0.051 (0.0387, 0.0661)
female 30–39	0.0513 (0.0349, 0.0728)	0.0342 (0.0232, 0.0485)	0.0257 (0.0174, 0.0364)	0.0552 (0.0375, 0.0784)	0.0368 (0.025, 0.0523)	0.0276 (0.0188, 0.0392)
male 40–49	0.243 (0.209, 0.28)	0.162 (0.139, 0.187)	0.121 (0.105, 0.14)	0.261 (0.225, 0.301)	0.174 (0.15, 0.201)	0.131 (0.113, 0.151)
female 40–49	0.132 (0.108, 0.161)	0.0881 (0.0717, 0.107)	0.0661 (0.0538, 0.0804)	0.142 (0.116, 0.173)	0.0949 (0.0772, 0.115)	0.0712 (0.0579, 0.0866)
male 50–59	0.915 (0.844, 0.989)	0.61 (0.563, 0.66)	0.457 (0.422, 0.495)	0.985 (0.909, 1.07)	0.656 (0.606, 0.71)	0.492 (0.454, 0.533)
female 50–59	0.367 (0.323, 0.415)	0.244 (0.215, 0.277)	0.183 (0.161, 0.207)	0.395 (0.348, 0.447)	0.263 (0.232, 0.298)	0.197 (0.174, 0.223)
male 60–69	3.43 (3.28, 3.6)	2.29 (2.18, 2.4)	1.72 (1.64, 1.8)	3.7 (3.53, 3.87)	2.47 (2.35, 2.58)	1.85 (1.76, 1.94)
female 60–69	1.32 (1.23, 1.42)	0.881 (0.817, 0.948)	0.66 (0.613, 0.711)	1.42 (1.32, 1.53)	0.948 (0.88, 1.02)	0.711 (0.66, 0.765)
male 70–79	13 (12.7, 13.4)	8.68 (8.44, 8.92)	6.51 (6.32, 6.69)	14 (13.6, 14.4)	9.34 (9.08, 9.6)	7.01 (6.81, 7.21)
female 70–79	5.03 (4.83, 5.25)	3.36 (3.22, 3.5)	2.52 (2.41, 2.63)	5.42 (5.2, 5.65)	3.61 (3.46, 3.77)	2.71 (2.6, 2.83)
male 80+	41 (40.3, 41.7)	27.3 (26.8, 27.8)	20.5 (20.1, 20.9)	44.1 (43.4, 44.8)	29.4 (28.9, 29.9)	22.1 (21.7, 22.5)
female 80+	24.2 (23.8, 24.7)	16.1 (15.8, 16.5)	12.1 (11.9, 12.4)	26.1 (25.6, 26.6)	17.4 (17.1, 17.7)	13 (12.8, 13.3)

## Discussion

In a newly emerging infectious disease like COVID-19 data are assembled in challenging circumstances that may contribute to the underestimation of cases and deaths. Data available on the total confirmed cases and deaths in Spain do not provide age and gender information. Here, we have obtained estimates by population group by multiplying the total confirmed cases and deaths by the proportions occurring in each group of a sample with that information. This is a limitation of our study since it is possible that the sample with demographic information may not be representative of the whole population.

We have seen that the approach of estimating crude CFRs by dividing the total number of deaths by the total number of confirmed cases produce results that are difficult to interpret due to several biases. For example, the estimated rates may overstate the true rates due to preferential inclusion of severe cases since data assembled during emergency settings typically contain people who seek medical care, have the most severe symptoms, and experience fatal outcomes. Following Verity *et al.* ^6^ we have adjusted by preferential ascertainment of severe cases by assuming complete ascertainment in the group with the highest attack rates, and using demography-adjusted under-ascertainment rates to estimate the number of infected individuals in each population group. In addition, CFRs may also be biased due to the delay between disease onset and death. We have adjusted for this bias by considering a specific distribution for the time from disease onset to death. These are strong assumptions that could greatly affect the results. We conducted a sensitivity analysis where we calculated the CFRs using different levels of ascertainment and different distributions for the time from disease onset to death. The sensitivity analysis yielded different values for the CFRs, and in all scenarios CFRs were higher in older age groups and males.

In addition, CFRs calculated in the initial phase of an epidemic are highly dependant of the point in time they are calculated. Here we provide estimates calculated with data from 14 May but rates calculated at a later point in time could be different.

## Conclusions

The assessment of the severity of COVID-19 and the determinants of its variation is essential for planning health resources and the design of mitigation policies, including intelligent strategies to release population from confinement while protecting the most vulnerable. In this article we have estimated CFRs by age group and gender in Spain accounting for censoring and ascertainment biases. We have found that COVID-19 is highly influenced by age and gender with higher rates in older ages and males. The procedures used to obtain the CFR estimates require strong assumptions and although the interpretation of their magnitude should be treated with caution, the differences observed by age and gender are fundamental underpinnings to inform decision-making.

## Data availability

### Source data

Data on total confirmed cases and deaths, as well as confirmed cases and deaths by age group and gender from a subset of the population are reported by the Spanish Ministry of Health and provided by 5. Population data for Spain are obtained from the National Institute of Statistics of Spain ^4^.

### Underlying data

Data used is available from GitHub ( https://github.com/Paula-Moraga/coronavirus-cfr) and archived with Zenodo ^12^.

Zenodo: Paula-Moraga/coronavirus-cfr: First release. http://doi.org/10.5281/zenodo.3856444 ^12^

This project contains the following underlying data:

- ccaa_covid19_casos2020-05-14.csv (number of confirmed cases in each of the 17 regions of Spain from 2020-02-21 to 2020-05-14)
- ccaa_covid19_fallecidos2020-05-14.csv (number of deaths in each of the 17 regions of Spain from 2020-03-03 to 2020-05-14)
- nacional_covid19_rango_edad2020-05-14.csv (number of confirmed cases, hospitalized, uci, and deaths in Spain for each age group and gender from 2020-03-23 to 2020-5-14)
- popspainagegroupsex1Jul19.csv (Spanish population for each age and gender in 2019)

Data are available under the terms of the Creative Commons Attribution 4.0 International license (CC-BY 4.0).

## Software availability

Code for the results, figures and tables of this study can be found at https://github.com/Paula-Moraga/coronavirus-cfr

Archived code at time of publication: http://doi.org/10.5281/zenodo.3856444 ^12^.

License: Creative Commons Attribution 4.0 International license
