# Supplementary material for: Assessing the age- and gender-dependence of the severity and case fatality rates of COVID-19 disease in Spain
Source: Wellcome Open Res. 2020 Jun 2;5:117. [Version 1] doi: 10.12688/wellcomeopenres.15996.1 (PMC8063525; doi:10.12688/wellcomeopenres.15996.1)
Supplement: Peer review history

# Peer review: Reviewer response for version 1

**DOI:** 10.21956/wellcomeopenres.17545.r43266

**Published:** 2021-04-13

**Authors:** George Jó Bezerra Sousa

**Affiliations:** 1 Universidade Estadual do Ceará, Fortaleza, Brazil

The article “Assessing the age- and gender-dependence of the severity and case fatality rates of COVID-19 disease in Spain” aims to calculate crude and adjusted fatality rates by age group and gender in Spain. Overall, the manuscript is well written with interesting analysis and important results. Minor considerations are given below.

1. In the introduction, the part from “while people of any age” to “in these groups” needs a reference.
2. Once the authors decided to go through a methodological approach on the use of models to calculate CFR in the COVID-19 scenario, the epidemiological features of the cases are not well addressed. It is understood that doing this type of analysis during an ongoing pandemic has its limitations. Then, I suggest addressing them as some limitations of the study.
3. The methods are well-stated and results are clearly presented.
4. Code and data files are available to download and replicate the study.

Is the work clearly and accurately presented and does it cite the current literature?

Yes

If applicable, is the statistical analysis and its interpretation appropriate?

Yes

Are all the source data underlying the results available to ensure full reproducibility?

Yes

Is the study design appropriate and is the work technically sound?

Yes

Are the conclusions drawn adequately supported by the results?

Yes

Are sufficient details of methods and analysis provided to allow replication by others?

Yes

Reviewer Expertise:

Infectious Diseases Epidemiology

I confirm that I have read this submission and believe that I have an appropriate level of expertise to confirm that it is of an acceptable scientific standard.

# Peer review: Reviewer response for version 1

**DOI:** 10.21956/wellcomeopenres.17545.r39137

**Published:** 2020-07-14

**Authors:** Timothy Russell

**Affiliations:** 1 Centre for Mathematical Modelling of Infectious Diseases, Department of Infectious Disease Epidemiology, London School of Hygiene and Tropical Medicine, London, UK

The authors calculated adjusted CFR estimates for Spain, adjusting directly for the delay between onset to death and indirectly for under-ascertainment and the age structure of the Spanish population. The adjustments for delay and for under-ascertainment require some assumptions, so the authors carry out a sensitivity analysis testing how different ascertainment assumptions and delay distribution assumptions affect the overall results. The sex and age-specific CFR estimates they calculate are consistent with other published CFR estimates and provide a useful and detailed contribution to the evidence base of COVID severity estimates.

 The discussion and application of the existing difficulties and biases when estimating many epidemiological parameters during an ongoing outbreak is lucid and well-written. It sheds light on the difficulties present, rather than glossing over them, which is all too often the case in other severity estimate papers.

 One minor comment is that the under-ascertainment adjustment includes some assumptions. Specifically, they assume that where cases are most severe (i.e. older age groups), case ascertainment is highest. A very fair assumption! But there are ascertainment estimates, in Verity *et al*. and many other detailed modelling papers (amongst others I'm sure), against which these assumptions could be tested. It is a minor comment, as the estimates are very much inline with other published estimates. But some discussion about the existence of more rigorous ascertainment estimates as a minimum requirement would help. Comparing such estimates to the assumed values would be even better.

 The code is available, but it would only run after some difficulty, as I believe some of the data loaded includes letter/accents specific to Spanish. It would not run on first attempt and I had to do quite a bit of digging and tweaking in the code to get it to run. I believe cleaning up the code so that it runs reproducibly and smoothly would also be a great benefit. However, the code is well written and commented and the analysis is relatively straight forward. It is great that it has been shared, as the analysis is almost reproducible just by reading the code to see what it does! Improving it would certainly help though.

 Apart from those minor points, this is a sound, interesting and well-written paper which addresses all of the difficulties in calculating CFRs during an outbreak and provides detailed estimates for Spain. I believe it would be of indexable standard if the code base is smoothed out a bit and some discussion of other case ascertainment estimates is included.

Is the work clearly and accurately presented and does it cite the current literature?

Yes

If applicable, is the statistical analysis and its interpretation appropriate?

Yes

Are all the source data underlying the results available to ensure full reproducibility?

Yes

Is the study design appropriate and is the work technically sound?

Yes

Are the conclusions drawn adequately supported by the results?

Yes

Are sufficient details of methods and analysis provided to allow replication by others?

Partly

Reviewer Expertise:

Mathematical modelling of infectious diseases.

I confirm that I have read this submission and believe that I have an appropriate level of expertise to confirm that it is of an acceptable scientific standard.
